# Bone Age Practices in Infants and Older Children among Practicing Radiologists in Pakistan: Developing World Perspective

**DOI:** 10.7759/cureus.3936

**Published:** 2019-01-22

**Authors:** Waseem A Mirza, Mukhtiar Memon, Sayed Mustafa Mahmood Shah, Umm e Laila Hussain, Arif Ali, Nauman Hashmani, Naila Nadeem, Muhammad Nadeem Ahmad

**Affiliations:** 1 Radiology, Aga Khan University Hospital, Karachi, PAK; 2 Radiology, Dow University of Health Science, Karachi, PAK; 3 Internal Medicine, Dow University of Health Sciences, Karachi, PAK; 4 Internal Medicine, Dow University of Health Science, Karachi, PAK; 5 Ophthalmology, Hashmanis Hospital, Karachi, PAK

**Keywords:** bone age, x-rays

## Abstract

Objective

To investigate which bone age assessment techniques are utilized by radiologists in Pakistan to determine skeletal age in three defined age groups: less than one year, one to three years and three to 18 years. We also assessed the perceived confidence in skeletal age assessments made by respondents using their chosen bone age assessment technique, within each defined age group.

Materials and methods

A cross-sectional survey was conducted among 147 practicing radiologists in Pakistan. A pre-validated survey form was adopted from a similar study conducted amongst members of the Society for Pediatric Radiology. The survey collected demographic information, choice of bone age assessment technique in each age group and confidence of bone age assessments in each age group.

Results

The hand-wrist method of Greulich and Pyle was used by 87.5% of respondents when assessing bone age in infants (less than one year), followed by Gilsanz-Ratib hand bone age method (7.3%). In children aged one to three years, Greulich and Pyle method was chosen by 85.7% of respondents, followed by Gilsanz-Ratib hand bone age method (6.1%) and the Hoerr, Pyle, Francis' Radiographic Atlas of Skeletal Development of the Foot and Ankle (3.1%). In children, older than three years, the Greulich and Pyle technique was used by 83.7% of respondents. This was followed by Gilsanz-Ratib hand bone age method (5.8%) and the Hoerr, Pyle, Francis' Radiographic Atlas of Skeletal Development of the Foot and Ankle (3.8%). 26.4% were “very confident” in bone age assessments conducted among infants. In children aged one to three years, 38.1% were “very confident”. In children, greater than three years, 48.6% were “very confident” in their chosen technique.

Conclusion

Greulich and Pyle is the dominant method for bone age assessments in all age groups, however, confidence in its application among infants and young children is low. It is recommended that clear recommendations be developed for bone age assessments in this age group alongside incorporation of indigenous standards of bone age assessments based on a representative sample of healthy native children.

## Introduction

Assessment of skeletal age using radiographic analysis is widely utilized for determination of age worldwide. Accurate assessment of skeletal age plays an indispensable role in the diagnosis of diseases resulting in tall or short stature, and measuring the response to treatment in such disorders [1]. Skeletal age assessment also plays an essential role in medico-legal cases [2], competitive sports and immigration. This is particularly important in a South-Asian setting where systematic registration of birth data is not uniformly present [3]. Bone age assessment (BAA) may be conducted using visualisation of hands and wrists using plain radiographs, following which images are qualitatively matched to reference images provided in a standard radiological atlas. The Greulich and Pyle (GP) radiographic atlas of the hand and wrist is one of the oldest and most commonly utilized atlas for this purpose worldwide [4, 5]. Gilsanz and Ratibin (GR) atlas also provides a bone age assessment scheme using detailed images of ossification centres in hand radiographs of healthy children and shows comparable accuracy to GP method [6]. Other methods such as Girdany and Golden (GG) method utilize a plain radiograph of various large joints of the body and determine skeletal age based upon the appearance of ossification centres around those joints [7].

Data regarding bone age practices from the developing world is limited. We conducted this study to investigate which bone age assessment techniques are primarily utilized by radiologists in Pakistan, and their perceived confidence in the use of their chosen techniques among various age groups.

## Materials and methods

We utilized a pre-validated 10-question survey item from a study conducted among members of Society for Pediatric Radiology (SPR) which mainly consisted of paediatric radiologists in North America (permission to use the survey form was granted by the study authors) [8]. The survey gathered basic demographic information regarding years of practice as a radiologist and frequency of bone age assessments by respondents in one week. The respondents were then inquired regarding the methodology applied in conducting BAA in the following age groups: children less than one year old, children one to three years old, and children aged from three to 18 years. Confidence in assessments made within each age group was also assessed. Further inquiry regarding the use of determination of bone in children with delayed skeletal maturity and reconciling bone age assessments which fall between two different atlas methods, were also conducted.

The survey was self-administered to a convenient sample of radiologists attending the annual conference of Radiological Society of Pakistan at Karachi, the largest national radiological event having representation from all over the country. Participation was voluntary and informed consent was acquired from all willing participants. The survey was conducted over a three-day period from 27-29th October 2017.

## Results

Demographics

**Figure 1:**
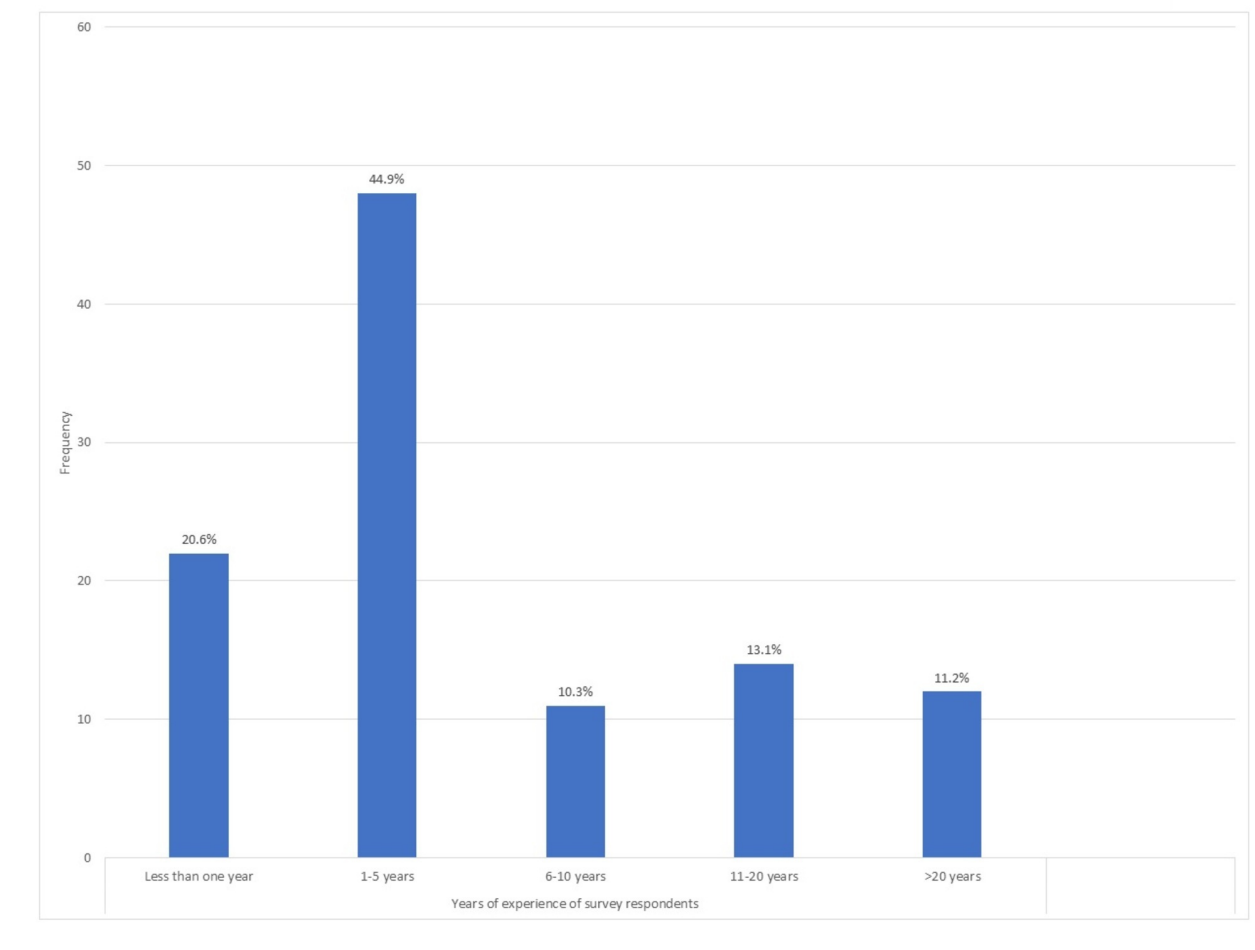
Years of experience of survey respondents.

The survey was self-administered to 186 radiologists attending the Pakistan Radiology Society Conference. Of these 147 opted to participate in the survey (response rate of 79.0%). Following the removal of incomplete forms, we were left with 107 response sheets for statistical analysis. Most of our study respondents reported one to five years of experience as a practicing radiologist (44.9%) (Figure 1).

**Figure 2:**
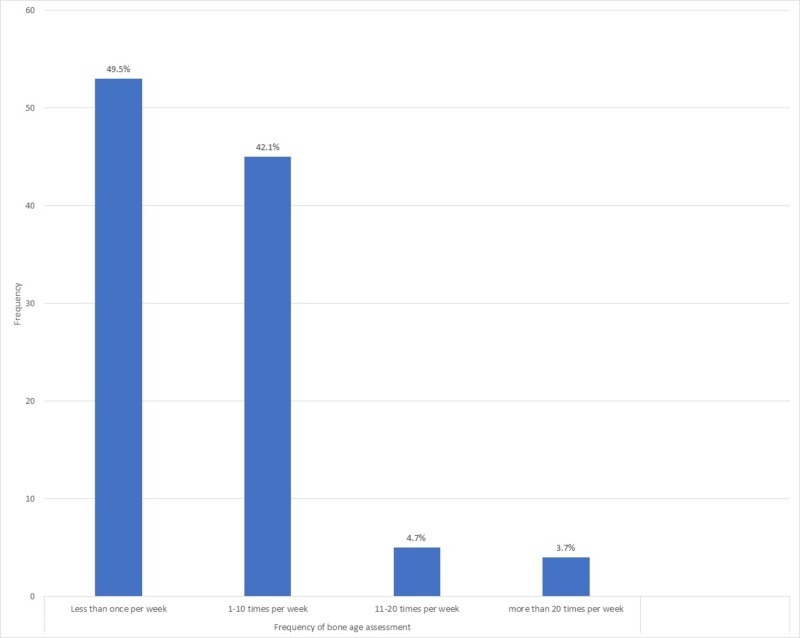
Frequency of bone age assessment.

A significant proportion of respondents reported less than one year of experience (20.6%). There was a decreasing trend in the frequency of bone age assessments conducted by our respondents, with 49.5% of respondents conducting bone age assessments less than one time a week. Only 8.4% reported conducting bone age assessments more than 11 times a week (Figure 2).

Practice patterns

**Figure 3:**
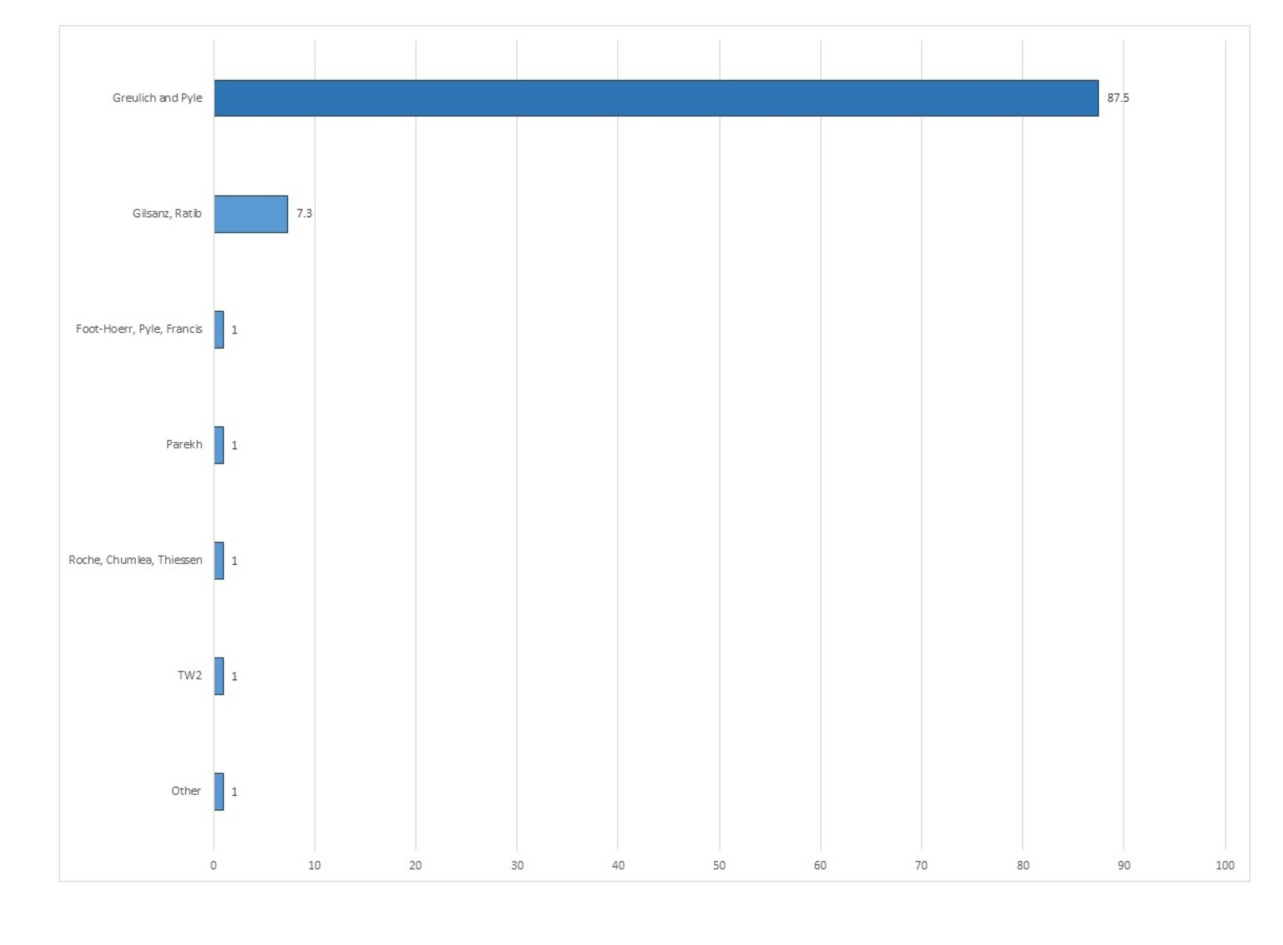
Distribution of bone age techniques used in infants younger than one year.

The hand-wrist method of Greulich and Pyle was the dominant bone age assessment method chosen for conducting bone age assessments in infants (87.5%) (Figure 3). This was followed by Gilsanz-Ratib hand bone age assessment method, chosen by 7.3% of respondents.

**Figure 4:**
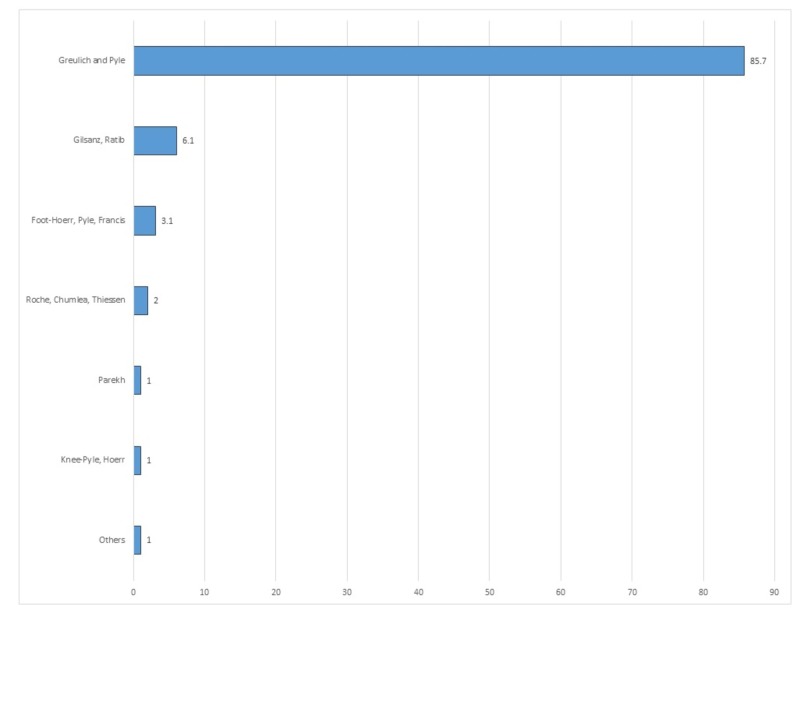
Distribution of bone age techniques used in children aged one to three years.

Among children aged one to three years (Figure 4), Greulich and Pyle remained the dominant bone age assessment scheme (85.7%), followed by Gilsanz-Ratib hand bone age method (6.1%) and the Hoerr, Pyle, Francis' Radiographic Atlas of Skeletal Development of the Foot and Ankle (3.1%).

**Figure 5:**
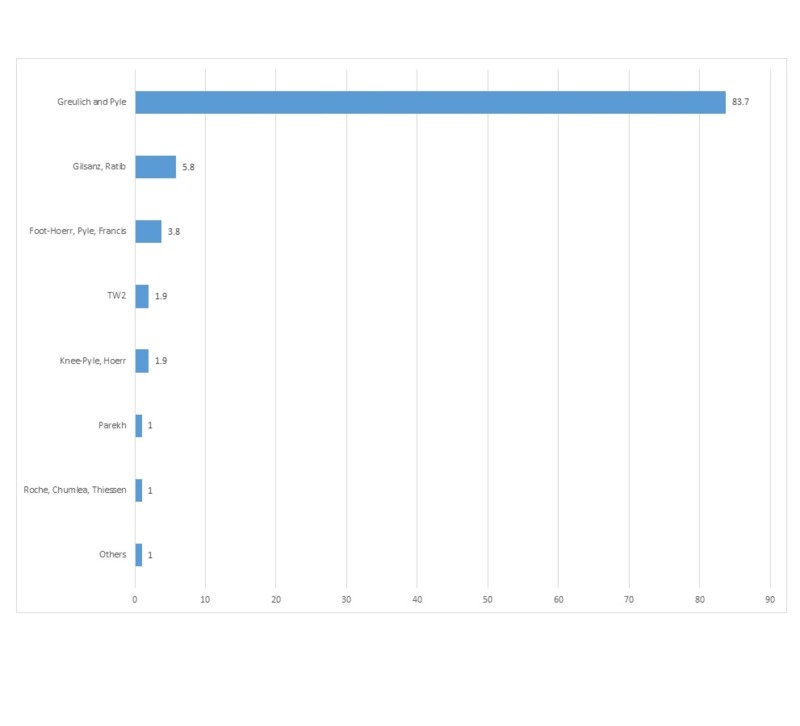
Distribution of bone age techniques used in children older than three years.

For children older than three years (Figure 5), the Greulich and Pyle technique was used by 83.7% of respondents. This was followed by Gilsanz-Ratib hand bone age method (5.8%) and the Hoerr, Pyle, Francis' Radiographic Atlas of Skeletal Development of the Foot and Ankle (3.8%).

When a bone age was substantially delayed compared to the chronological age, only 12.7% of respondents “frequently” opted to utilize a different BAA technique. 43.1% opted for a different technique under such circumstances “occasionally”, and 44.1% “never” opted for an alternative technique. When the estimated bone age assessment fell between two standards, 39.8% of respondents opted to provide the range of the two reference standards, while 37.6% opted to pick one of the two standards. Only 12.9% opted to provide a mid-point interpolation between two reference standards under such circumstances.

Reader confidence

**Figure 6:**
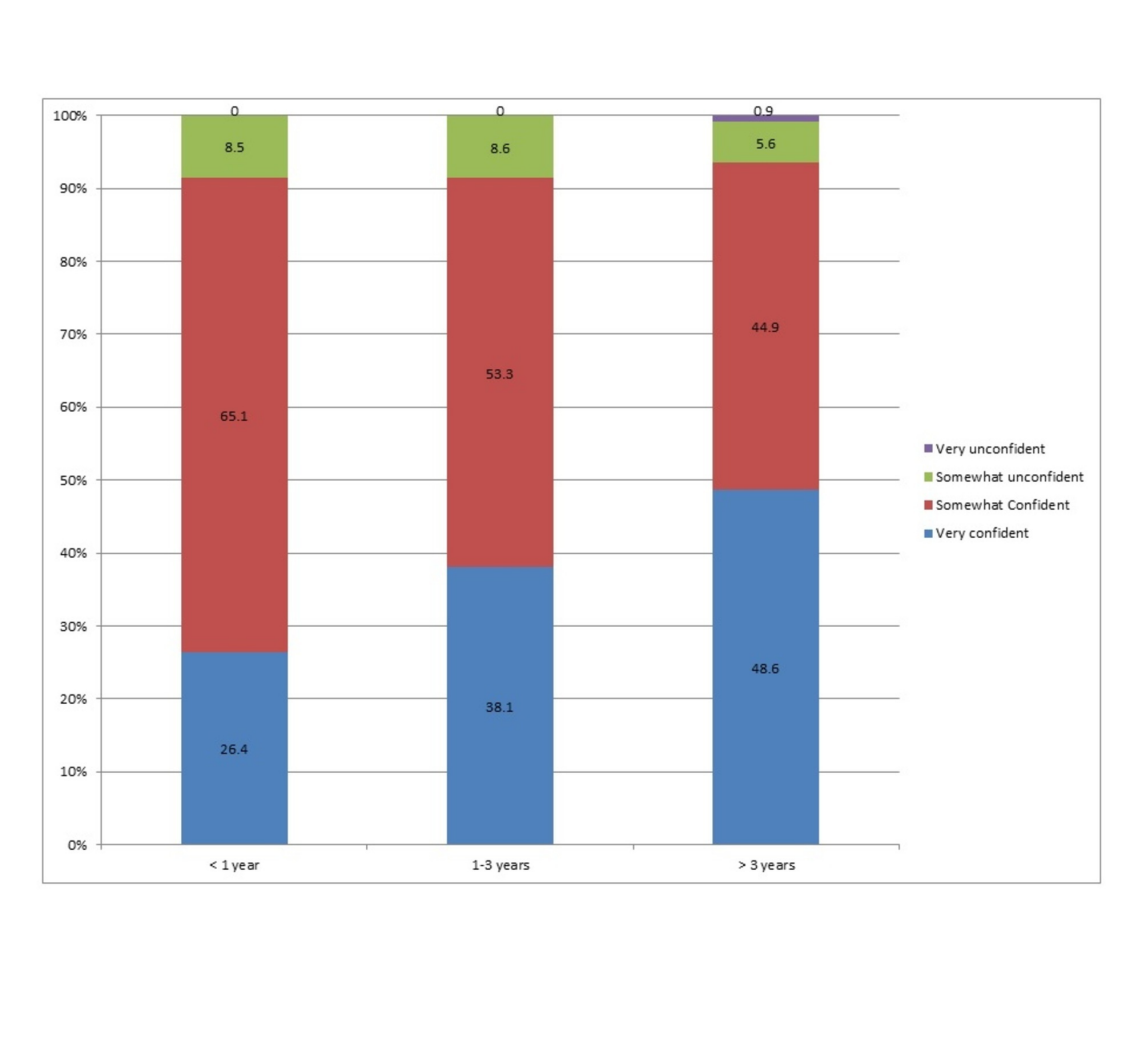
Radiologists’ confidence levels in chosen bone age assessment techniques in the three age groups.

In infants, 91.5% of respondents expressed confidence in their selected technique of bone age assessment, of which 26.4% were “very confident”. In children aged one to three years, 91.4% of respondents expressed confidence in their bone age technique, of which 38.1% were “very confident”. In children, greater than three years, 93.5% reported confidence in their chosen bone age assessment technique, of which 48.6% were very confident (Figure 6).

## Discussion

Our results show that the GP radiologic atlas of the hand and wrist is the most popular BAA technique among radiologists practicing in Pakistan. The GP atlas is one of the oldest and most popular BAA schemes worldwide [3], and has been validated among various ethnic groups [9]. However, studies conducted among the South-Asian paediatric population have shown limitations in the application of GP atlas. A cross-sectional study conducted in Karachi, Pakistan, among a random sample of children aged 4.5 to 9.5 years, showed the GP atlas underestimates chronological age by 6.65 ± 13.47 months in females and 15.78 ± 12.83 months in males [3]. A second study in Karachi, conducted among children aged up to 18 years confirmed that GP was unable to accurately assess skeletal age in boys, although measurements in girls were deemed accurate [4]. A similar study conducted in India concluded that GP atlas is not applicable to the Indian children of both sexes especially in middle and late childhood [10]. The popularity of GP atlas in our setting may be attributed to its simplicity and greater uptake among general radiologists. This is in contrast to other BAA schemes such as Tanner Whitehouse 2 (TW2) which are more complex and require more time [3].

Our results showed that radiologists practicing in Pakistan expressed decreasing levels of confidence in the accuracy of their chosen bone age assessment techniques in younger age groups. This is consistent with a similar survey conducted among paediatric radiologists in America, where 34% of respondents lacked confidence when assessing bone age in infants [8]. The rapid skeletal growth occurring in infants makes accurate bone age assessment difficult in this sub-group. Alternative methods for bone age assessment in this subgroup have been proposed including bone age estimation based on fibular shaft length [11] and use of an ultrasonographic version of the GP atlas [12]. Recently a new, quantitative method for bone age assessment using capito-hamate (CH) planimetry (defined as the measurement of the sum of areas of the capitate and hamate) has been proposed. This method has shown a strong positive correlation between chronological age and CH planimetry measurements. No significant difference in accuracy between CH planimetry and the GP method was observed, however inter-observer reproducibility of CH planimetry was greater than that of the GP method.

Our study is subject to certain limitations. This was a cross-sectional survey conducted among a convenience sample of radiologists attending the conference, as such our results may not be applicable to all practicing radiologists in Pakistan. Our study did not involve an objective measurement of a radiologist’s confidence in their chosen method of BAA, as such there is a risk of social desirability bias. Lastly, our survey was not limited to paediatric radiologists. This is because there is a dearth of qualified paediatric radiologists in our country, due to which bone age assessments are frequently performed by general radiologists as well.

## Conclusions

Greulich and Pyle is the dominant method for BAA among radiologists practicing in Pakistan. Confidence in BAA is high among older children, but lacking in infants (less than one year). It is recommended that indigenous standards of bone age assessments be developed based on a representative sample of healthy native children.
